# The* Type 2 Deiodinase Thr92Ala Polymorphism* Is Associated with Worse Glycemic Control in Patients with Type 2 Diabetes Mellitus: A Systematic Review and Meta-Analysis

**DOI:** 10.1155/2016/5928726

**Published:** 2016-09-29

**Authors:** Xiaowen Zhang, Jie Sun, Wenqing Han, Yaqiu Jiang, Shiqiao Peng, Zhongyan Shan, Weiping Teng

**Affiliations:** The Endocrine Institute and The Liaoning Provincial Key Laboratory of Endocrine Diseases, Department of Endocrinology and Metabolism, The First Hospital of China Medical University, No. 155 Nanjing North Street, Shenyang, Liaoning, China

## Abstract

*Objective*. Type 2 deiodinase (Dio2) is an enzyme responsible for the conversion of T4 to T3. The Thr92Ala polymorphism has been shown related to an increased risk for developing type 2 diabetes mellitus (T2DM). The aim of this study is to assess the association between this polymorphism and glycemic control in T2DM patients as marked by the HbA1C levels.* Design and Methods.* The terms “rs225014,” “thr92ala,” “T92A,” or “dio2 a/g” were used to search for eligible studies in the PubMed, Embase, and Cochrane databases and Google Scholar. A systematic review and meta-analysis of studies including both polymorphism testing and glycated hemoglobin (HbA1C) assays were performed.* Results*. Four studies were selected, totaling 2190 subjects. The pooled mean difference of the studies was 0.48% (95% CI, 0.18–0.77%), indicating that type 2 diabetics homozygous for the Dio2 Thr92Ala polymorphism had higher HbA1C levels.* Conclusions*. Homozygosity for the Dio2 Thr92Ala polymorphism is associated with higher HbA1C levels in T2DM patients. To confirm this conclusion, more studies of larger populations are needed.

## 1. Introduction

*Type 2 deiodinase (Dio2)* is localized on the long arm of the 14th human chromosome in 14q24.3. It is an intracellular enzyme which catalyzes the conversion of thyroxine (T4) to its active form triiodothyronine (T3) [1]. Therefore, Dio2 is a very important regulator for tissue specific metabolic activity.

A single nucleotide polymorphism in the* Dio2* gene (A/G) results in a threonine change to alanine (Thr92Ala) at codon 92 [2]. The* Dio2* Thr92Ala polymorphism has been reported to be associated with many disorders, such as osteoarthritis [3, 4], hypertension [5], Graves' disease [6], Kashin-Beck disease [7], bipolar disorder [8], depression [9], and cognitive impairment [10, 11]. In addition, many studies have shown that the Thr92Ala polymorphism is related to type 2 diabetes mellitus (T2DM), insulin resistance, and body mass index (BMI) [2, 12–18]. However, the relationship between the Thr92Ala polymorphism and glycemic control in T2DM patients is unclear. The aim of this meta-analysis is to determine whether the* Dio2* Thr92Ala polymorphism is associated with glycemic control in T2DM patients as marked by the HbA1C level.

## 2. Design and Methods

### 2.1. Search Strategy

A literature search was conducted by two investigators (Xiaowen Zhang and Jie Sun) in February 2016, using the PubMed, Embase, and Cochrane databases and Google Scholar. The terms Thr92Ala, T92A, rs225014, and Dio2 a/g were used. Reference lists in the articles and recent reviews were also searched.

### 2.2. Inclusion Criteria

Studies were selected for meta-analysis if they met the following criteria: (1) being observational studies related to* Dio2* polymorphism and (2) including T2DM patients along with their respective glycated hemoglobin (HbA1C) levels.

### 2.3. Data Extraction

The following data were extracted from selected studies: name of first author, year of publication, country, age, gender, duration of diabetes, treatment, population size, and HbA1C means and standard deviations (SD) of each genotype. The difference between genotypes was analyzed in both recessive and dominant inheritance model.

### 2.4. Quality Assessment

Two investigators (Yaqiu Jiang and Wenqing Han) assessed the quality of the selected studies using the Newcastle-Ottawa Scales (NOS) [19]. Information regarding selection, comparability, and exposure was evaluated for each study. Only the studies with more than 5 stars based on the NOS scale were included in this meta-analysis.

### 2.5. Statistical Analyses

This systematic review was conducted according to the recommendations outlined in the PRISMA (Supplementary Material available online at http://dx.doi.org/10.1155/2016/5928726). Review Manager 5.3 was used for data analysis. The mean differences and the 95% confidence intervals (95% CI) for each study and for the pooled effect were calculated to evaluate the relationship between Thr92Ala polymorphism and HbA1C value in T2DM patients. Heterogeneity was assessed using the *Q* statistical test and *I* ^2^ statistic. A sensitivity analysis was performed by sequential omission of each study. Subgroup analysis was processed to investigate heterogeneity in the meta-analysis.

## 3. Results

**Figure 1:**
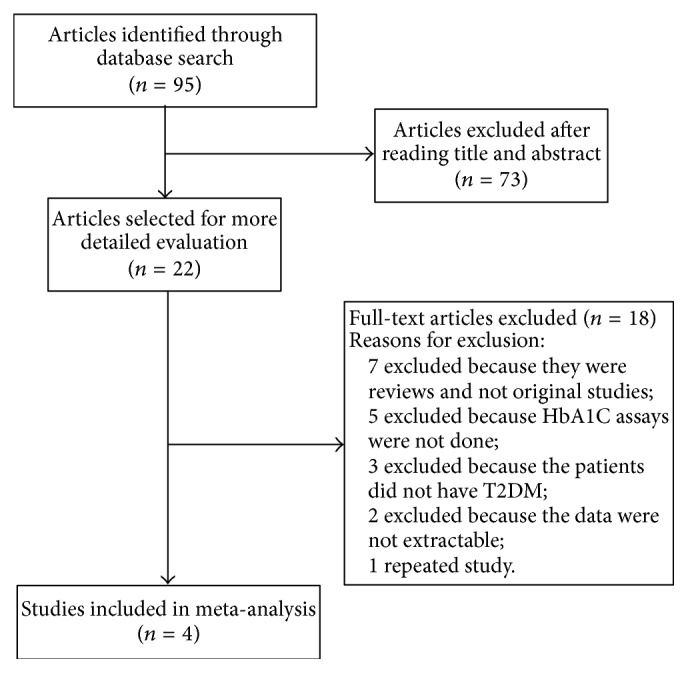
Flowchart of search results for meta-analysis.

Ninety-five studies were initially retrieved from the databases and Google Scholar. Seventy-three articles were excluded after reading the titles and abstracts as they were not related to T2DM. A full-text review of the remaining 22 articles was conducted and 4 articles were identified as eligible (Figure 1).  Table 1 summarized the characteristics of the selected articles.

**Figure 2:**
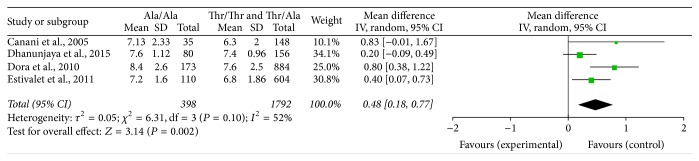
An analysis in recessive inheritance model. Individual and pooled MDs and 95% Cl estimate for HbA1C levels in type 2 diabetes in association with Thr92Ala homozygosity of type 2 deiodinase. CI: confidence interval; HbA1C: glycated hemoglobin; MD, mean differences.

When analyzed in a recessive inheritance model, the meta-analysis of 4 studies including data on the association between Thr92Ala polymorphism and HbA1C levels showed that the mean difference (MD) was 0.48% (95% CI, 0.18~0.77%) (Figure 2), indicating that the HbA1C levels in T2DM patients with* Dio2* Thr92Ala homozygosity are 0.48% higher than the non-Ala T2DM patients. Both the *Q* statistical test (*P* = 0.10) and *I* ^2^ statistic (*I* ^2^ = 52%) showed moderate heterogeneity; therefore, a random effects model was used. Sensitivity analyses by sequential omission of each study resulted in similar results, with calculated MDs of 0.44% (95% CI, 0.11~0.76%), 0.60% (95% CI, 0.30~0.89%), 0.33 (95% CI, 0.09~0.57%), and 0.55% (95% CI, 0.07~1.02%), respectively.

**Figure 3:**
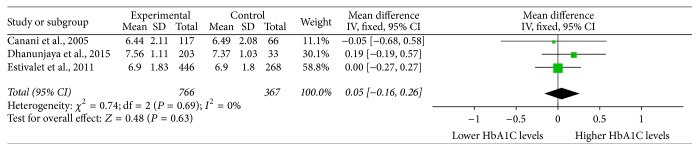
An analysis in dominant inheritance model. Individual and pooled MDs and 95% Cl estimate for HbA1C levels in type 2 diabetes in association with Thr92Ala homozygosity of type 2 deiodinase. CI: confidence interval; HbA1C: glycated hemoglobin; MD, mean differences.

When analyzed in a dominant inheritance model, one study was excluded because Thr/Thr and Ala/Thr genotypes were grouped and compared with Ala/Ala genotype [14]. The meta-analysis of the remaining 3 studies showed that the mean difference (MD) was 0.05% (95% CI, −0.16~0.26%) (Figure 3), indicating that HbA1C levels of T2DM patients with* Dio2* Thr92Ala allele were not higher. Both the *Q* statistical test (*P* = 0.69) and *I* ^2^ statistic (*I* ^2^ = 0%) showed no heterogeneity; therefore, a fixed effects model was used. Sensitivity analyses by sequential omission of each study resulted in similar results, with calculated MDs of 0.06% (95% CI, −0.16~0.29%), −0.01% (95% CI, −0.26~0.24%), and 0.13% (95% CI, −0.20~0.45%), respectively.

**Figure 4:**
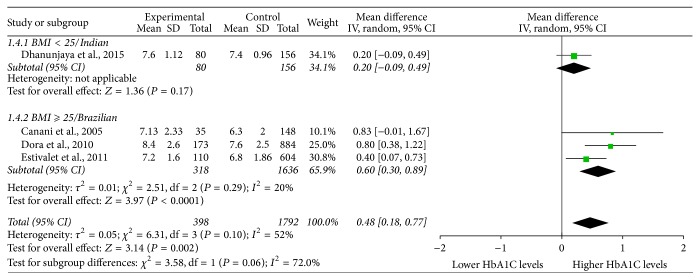
Subgroup analysis. Individual and pooled MDs and 95% Cl estimate for HbA1C levels in type 2 diabetes in association with Thr92Ala homozygosity of type 2 deiodinase. CI: confidence interval; HbA1C: glycated hemoglobin; MD, mean differences.

In order to uncover the source of heterogeneity, subgroup analysis was performed (Figure 4). Dhanunjaya's study is different from the other 3 studies because the BMI is smaller and racial population is Indian. We found that this study was the main source of heterogeneity.

## 4. Discussion

The results of the previous studies regarding the association between the* Dio2* Thr92Ala polymorphism and glycemic control in T2DM patients have been contradictory [2, 14, 20, 21]. This current meta-analysis found that people who are homozygous for Thr92Ala had 4.8% higher HbA1C levels, suggesting that Thr92Ala homozygosity is associated with worse glycemic control in T2DM patients. To our knowledge, this is the first meta-analysis to investigate the effect of* Dio2* Thr92Ala polymorphism on HbA1C levels in T2DM patients.

Some studies assumed that it is a recessive inheritance model because of “Thr/Thr and Thr/Ala genotypes showing similarities in biochemical characteristics” [14, 20]. However, we believe that further studies are still needed and analyses performed in dominant inheritance model are necessary. There are no significant differences in Ala/Ala + Ala/Thr versus Thr/Thr when it was analyzed in a dominant inheritance model.

Among the four included studies, three were conducted in Brazil, thus related to Brazilian population [2, 14, 21], and one was done in India [20]. In the Brazilian population, the Ala allele frequency is about 0.4; however, in the Indian population, the frequency of this allele is 0.6, which is much higher than that among the Brazilian population. It has been reported that the Ala allele frequencies vary among different populations: Indians (0.61), Han Chinese in Beijing (0.58), Mexican-Americans (0.54), African American (0.51), British (0.34), and Finnish (0.24) (https://www.ncbi.nlm.nih.gov/variation/tools/1000genomes/). Subgroup analysis shows that Dhanunjiya's study, which was different in BMI or race from the other 3 studies, would be the reason for moderate heterogeneity. Both BMI and race might have apotential impact on the HbA1C levels in T2DM patients. Further studies are needed to investigate these potential influential factors.

Carriers of the Ala allele have been shown to have a net decrease in glucose disposal [22]. The* Dio2* Thr92A and peroxisome* proliferator-activated receptor-gamma2* (PPAR*γ*2) Pro2Ala polymorphisms interact in the modulation of systolic and diastolic blood pressure and metabolic syndrome [23]. Furthermore, a combination of this polymorphism and*β3-adrenergic receptor* Trp63Arg results in an increased BMI, indicating a synergistic effect of these two polymorphisms [13].

Some studies have shown that the Thr92Ala polymorphism is related to type 2 diabetes mellitus (T2DM), insulin resistance. However, the Framingham Heart Study showed that there is no association of Thr92Ala polymorphism with T2DM or hypertension risks [17, 24]. Though Dio2 velocity was decreased in the thyroid and skeletal muscle in individuals homozygous for the Ala allele [2], the Thr to Ala substitution at codon 92 is not near the enzyme's active site [25]. Moreover, the Thr92Ala mutation does not affect Dio2 activity* in vitro*, indicating that Thr92Ala may be a marker, rather than a functional polymorphism of the decreased enzyme velocity.

The limitations of this meta-analysis are as follows: (1) T2DM is a heterogeneous disease [26], and thus the contribution of a single gene may be restricted; (2) the size of subjects involved in this meta-analysis was relatively small; (3) only Brazilian and Indian population were included and these findings may not be applied to other racial groups. More studies on this topic are needed since genetic association studies should include larger populations in order to ensure accurate conclusions.

## 5. Conclusion

In conclusion, homozygosity for the* Dio2* Thr92Ala polymorphism might be associated with worse glycemic control in type 2 diabetics as marked by the HbA1C levels. Further epidemiological studies of larger populations from different geographic regions are needed.

## Supplementary Material

This review protocol has been prepared according to the Preferred Reporting Items for Systematic reviews and Meta-Analysis (PRISMA). From: Moher D, Liberati A, Tetzlaff J, Altman DG, The PRISMA Group (2009). Preferred Reporting Items for Systematic Reviews and Meta-Analyses: The PRISMA Statement. PLoS Med 6(6): e1000097. doi:10.1371/journal.pmed1000097

## Figures and Tables

**Table 1:** Characteristics of selected studies.

Author name	Year	Country	DM	Treatment	Ala/Ala subjects (*n*)			Non-Ala/Ala subjects (*n*)			Ala allele frequency	NOS scale
			Duration (year)		*N* (%)	Age	Male (%)	*N* (%)	Age	Male (%)		
Canani	2005	Brazil	11.4 ± 8.6	Diet alone/anti-DM drugs	35 (19.1)	58.0 ± 9.8	45.7	148 (80.9)	58.1 ± 10.9	42.6	0.41	7
Dora	2010	Brazil	10 (5–17)	Insulin/anti-DM drugs	173 (16.4)	46.9 ± 10.7	43.4	884 (83.6)	47.5 ± 11.0	48.0	0.38	7
Dhanunjaya	2015	India	NS	NS	80 (33.9)	60.0 ± 9.0	100.0	156 (66.1)	57.0 ± 10	100.0	0.60	6
Estivalet	2011	Brazil	12.1 ± 9.4	Diet alone/anti-DM drugs/insulin & anti-DM drugs	110 (15.4)	58.3 ± 9.4	41.4	604 (84.6)	60.7 ± 9.7	48.9	0.39	7
